# Dietary and Antioxidant Vitamins Limit the DNA Damage Mediated by Oxidative Stress in the Mother–Newborn Binomial

**DOI:** 10.3390/life12071012

**Published:** 2022-07-08

**Authors:** Hector Diaz-Garcia, Jenny Vilchis-Gil, Pilar Garcia-Roca, Miguel Klünder-Klünder, Jacqueline Gomez-Lopez, Javier T. Granados-Riveron, Rocio Sanchez-Urbina

**Affiliations:** 1 Unidad de Investigación en Malformaciones Congénitas, Hospital Infantil de Mexico Federico Gómez, Mexico City 06720, Mexico; hecdiazgar@gmail.com (H.D.-G.); javiertgranados@gmail.com (J.T.G.-R.); 2 Sección de Estudios de Posgrado e Investigación de la Escuela Superior de Medicina del Instituto Politécnico Nacional, Mexico City 11340, Mexico; 3 Unidad de Investigación Epidemiológica en Endocrinología y Nutrición, Hospital Infantil de Mexico Federico Gómez, Mexico City 06720, Mexico; jvilchis@himfg.edu.mx; 4 Facultad de Medicina, Universidad Nacional Autónoma de Mexico, Mexico City 04510, Mexico; 5 Unidad de Investigación y Diagnóstico en Nefrología y Metabolismo Mineral Ósea, Hospital Infantil de Mexico Federico Gómez, Mexico City 06720, Mexico; mipgr5910@gmail.com; 6 Subdirección de la Gestión de la Investigación, Hospital Infantil de Mexico Federico Gómez, Mexico City 06720, Mexico; klunderk@gmail.com; 7 Hospital Militar de Especialidades de la Mujer y Neonatología, Secretaria de la Defensa Nacional, Mexico City 11200, Mexico; drajackiegomezlopez@yahoo.com.mx; 8 Escuela Superior de Medicina del Instituto Politécnico Nacional, Mexico City 11340, Mexico

**Keywords:** pregnancy, vitamin A, 8-hydroxy-2′-deoxyguanosine, DNA damage, oxidative stress

## Abstract

During pregnancy, appropriate nutritional support is necessary for the development of the foetus. Maternal nutrition might protect the foetus from toxic agents such as free radicals due to its antioxidant content. In this study, 90 mothers and their children were recruited. DNA damage mediated by oxidative stress (OS) was determined by the levels of 8-hidroxy-2′-deoxyguanosine (8-OHdG) in the plasma of women and umbilical cord blood. The mothers and newborns were categorised into tertiles according to their 8-OHdG levels for further comparison. No relevant clinical differences were observed in each group. A strong correlation was observed in the mother–newborn binomial for 8-OHdG levels (Rho = 0.694, *p* < 0.001). In the binomial, a lower level of 8-OHdG was associated with higher consumption of calories, carbohydrates, lipids, and vitamin A (*p* < 0.05). In addition, the levels of 8-OHdG were only significantly lower in newborns from mothers with a higher consumption of vitamin A and E (*p* < 0.01). These findings were confirmed by a significant negative correlation between the 8-OHdG levels of newborns and the maternal consumption of vitamins A and E, but not C (Rho = −0.445 (*p* < 0.001), −0.281 (*p* = 0.007), and −0.120 (*p* = 0.257), respectively). Multiple regression analysis showed that the 8-OHdG levels in mothers and newborns inversely correlated with vitamin A (β = −1.26 (*p* = 0.016) and −2.17 (*p* < 0.001), respectively) and pregestational body mass index (β = −1.04 (*p* = 0.007) and −0.977 (*p* = 0.008), respectively). In conclusion, maternal consumption of vitamins A and E, but not C, might protect newborns from DNA damage mediated by OS.

## 1. Introduction

Quality and quantity of diet depend on the interplay between complex factors, such as the environment, personal economics, cultural background, and food availability [1]. During pregnancy, nutritional changes take place to supply the demands of both the mother and the foetus, and they are important for the normal development of the foetus [2]. Besides the mother’s diet, the foetus is susceptible to damage by maternal diseases, such as type 2 diabetes (T2D), obesity [3,4,5], or smoking [6,7], and the consumption of alcohol [8,9]. Under these conditions, free radicals (FR) are overproduced and accumulate in cells.

FR are molecules with an unpaired electron in their orbitals, which they donate to biomolecules, such as proteins, lipids, or DNA, to stabilise their own structure [10] while altering the structure and function of the biomolecule. Oxidative stress (OS) is a physiological status characterised by an accumulation of FR due to an imbalance in their production and elimination [11]. OS represents a danger to the organs and tissues of the mother and the foetus, but the antioxidant enzymatic system (superoxide dismutase, glutathione peroxidase, catalase, etc.) and the non-enzymatic system (transferrin, lactoferrin, phytochemicals, vitamins, etc.) limit FR overproduction [12,13,14,15].

OS might cause foetus damage [16] or predispose the progeny to develop certain diseases during their lifetime, such as cardiovascular diseases, T2D, or cancer [17]. Maternal diet might be a factor that influences the predisposition of the progeny toward these diseases.

Among the nitrogenous bases of DNA, guanine is the most susceptible to oxidation by OS [18]. Guanine oxidation produces 8-hydroxy-2′-deoxyguanosine (8-OHdG), which is used as a marker for OS-mediated DNA damage [19]. Increased 8-OHdG is associated with the progression or prognosis of cardiovascular diseases, T2D [20,21], cancer [22,23], and other diseases in adults [24,25,26,27,28,29]. In pregnant women, high levels of 8-OHdG have been associated with restriction of foetal growth [30,31], neural tube defects [32], and orofacial clefts [33] in the progeny. On the other hand, in mothers, high 8-OHdG levels were associated with gestational diabetes mellitus (GDM) [34,35], and more recently, it was observed in pregnant women infected by severe acute respiratory syndrome coronavirus 2 [36].

Food antioxidants such as vitamins A, C, and E have been widely studied for their ability to regulate and maintain physiological levels of OS in the body [37]. These vitamins, when used in highly pro-oxidative conditions (smokers), decreased the levels of 8-OHdG [38]. However, there is limited information about the effect of the maternal diet on OS-mediated DNA damage in mother–newborn binomials. The aim of this study was to determine whether the consumption of calories, macronutrients, and the vitamins A, C, and E during pregnancy modified OS-mediated DNA damage at the end of pregnancy in both the mother and the child. In this study, there was a strong correlation between 8-OHdG levels in the mother–child binomial, and the intake of vitamin A during pregnancy decreased 8-OHdG levels in the mothers and their children.

## 2. Materials and Methods

### 2.1. Participants

This study was performed with 90 healthy women and their children. Participants were recruited between January 2016 and December 2017. All women signed an informed consent form. The study was conducted in accordance with the latest version of the Declaration of Helsinki, and the protocol was approved by the Ethics and Biosecurity committees of the Hospital Infantil de Mexico Federico Gómez. We included women who did not have any nutritional conditions before pregnancy, delivered 38 to 40 weeks from their last menstruation, and had adequate prenatal control, including monthly medical consultations from the first trimester of pregnancy and a minimum of two normal ultrasonography results. Women who had pre-eclampsia, type 1 diabetes, T2D, or GDM were excluded. Data on the weight of the women before pregnancy were obtained from their medical history record. A physician measured the heights of the women by using a stadiometer (SECA, Hamburg, Germany) at the time of recruitment. The anthropometric data on weight and length, gestational age, mode of delivery, and sex of newborns were registered at birth. Mothers were categorised according to their pregestational body mass index (BMI, kg/m^2^) into normal-weight, BMI 18.5–24.9; overweight, BMI 25–29.9; and obese, BMI 30 and above. The pregestational record of smoking (number of cigarettes per day and period during the pregnancy) and alcohol consumption were collected from responses to direct questions during the medical examination.

### 2.2. Frequency of Food Consumed

A food frequency questionnaire to assess regular food intake was administered by a trained doctor to all participants in the third trimester before labour, as previously described [39]. The questionnaire was validated for estimated folate intake in the Mexican population. Briefly, the amount of food consumed was calculated in terms of the unit of measurement used (i.e., piece, cup, plate, or spoon) and the size of the unit (i.e., small, medium, or large). The frequency of consumption was calculated in grams or millilitres per day. The participants’ daily intake of energy (calories per day), macronutrients (carbohydrates, lipids, and proteins), and vitamins (A, C, and E) were calculated using the Food Processor SQL programme (version 10.9.0, 2011; ESHA Research, Salem, Oregon) and Mexican food tables, including data on traditional Mexican food [40]. For the statistical analysis, missing data on vitamins (*n* = 2) and calorie values above 2.5 standard deviation (SD) (*n* = 3) were excluded.

### 2.3. Blood Samples

During the last medical examination before delivery, 5 mL peripheral blood was collected from the women. Immediately after birth, the umbilical cord was clamped, and 5 mL blood samples were collected from it. All blood samples were collected in ethylenediaminetetraacetic acid-containing tubes, centrifuged at 1800× *g*/15 min, and subjected to protein precipitation with 20% trichloroacetic acid (50:50 *v/v*). Then, centrifugation was repeated at 3600× *g*/5 min at 4 °C. The plasma was stored at −80 °C until 8-OHdG determination.

### 2.4. 8-OHdG Quantification

The 8-OHdG level was determined in the plasma by immunofluorescence using the 8-hydroxy-2′-deoxyguanosine enzyme immunoassay (EIA) kit (CEDERLANE, Burlington, NC, USA) as previously reported [41]. Standard curves were constructed starting with a bulk standard of 8-OHdG (30 ng/mL, 8-hydroxy-2-deoxy Guanosine EIA Standard, Cat# CL89120KC) and 8 dilutions (3000 pg/mL to 10.3 pg/mL), and the assays were carried out according to the manufacturer’s instructions. Briefly, 50 μL of plasma, the EIA buffer, the 8-OHdG standard, and the 8-OHdG-acetylcholinesterase tracer were added to the wells. The monoclonal antibody against 8-OHdG was added, and the plate was incubated for 18 h at 4 °C followed by several washes with wash buffer. Then, 200 μL of Ellman’s reagent was added to each well. Optical densities were determined at 405 nm.

### 2.5. Clinical and Nutrient Profiles Based on 8-OHdG Tertiles

**Figure 1:**
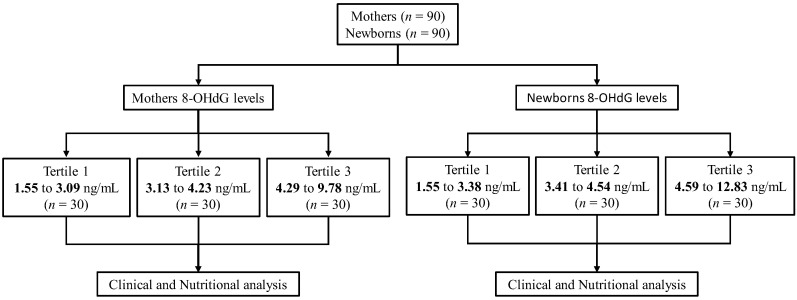
Group distribution by tertile of the 8-OHdG levels of mothers and newborns for nutritional analysis. 8-OHdG, 8-hydroxy-2′-deoxy-guanosine.

For the analysis, tertiles were constructed from the 8-OHdG values of the mothers and the newborns. For the mothers, tertile 1 was 1.55–3.09 ng/mL, tertile 2 was 3.13–4.23 ng/mL, and tertile 3 was 4.29–9.78 ng/mL, and for newborns, tertile 1 was 1.55–3.38 ng/mL, tertile 2 was 3.41–4.54 ng/mL, and tertile 3 was 4.59–12.83 ng/mL. Each tertile comprised 30 mothers and newborns (Figure 1). For micronutrients and macronutrients, the percentages of adequate intake were estimated using the recommended Dietary Reference Intakes from the Standing Committee On The Scientific Evaluation Of Dietary Reference Intakes (National Academy of Science, Washington, DC, USA) [42].

### 2.6. Statistical Analyses

Descriptive statistics, including mean, SD, median, interquartile range, and frequency, were used to describe the baseline characteristics of mothers and newborns. Medians and tertile ranges were used to report the daily calories obtained from the food consumed; amounts of proteins, carbohydrates, and fats; and the estimated consumption of vitamins A, C, and E. Based on a pre-exploratory variable analysis to identify their distributions, we used the Kruskal–Wallis rank sum test following a multiple comparison of p-values adjusted with the post-hoc Bonferroni method. Fisher’s exact test and the χ^2^ test were used to compare categorical variable differences. Quantile regression models were used to evaluate the association of clinical characteristics, macronutrients, and micronutrients with 8-OHdG levels. Data analysis and visualisation was performed in R version 3.6.2 (12 December 2019, Bell Laboratories, Murray Hill, NJ, USA) and STATA/SE 17.0 statistical programme (STATA Corp., College Station, TX, USA). *p*-values < 0.05 were considered to indicate statistically significant differences.

## 3. Results

### 3.1. Subject Characteristics

This study was performed with 90 women and their newborns. The mean age of the mothers was 24.1 ± 5.2 years, their mean pregestational weight was 63.8 ± 11.1 kg, and their mean pregestational BMI was 26.1 ± 4.0. Of them, 43.3% were normal-weight, 33.3% were overweight, and 23.3% were obese. Smoking during the first trimester of pregnancy was reported by 10% of the mothers, whereas only 1.1% (*n* = 1) reported alcohol consumption. Multivitamin supplements were consumed during pregnancy by 91.1% of women. The mean age of the newborns was 38.7 ± 1.3 weeks, their mean weight was 3188 ± 435 g, and their mean height was 49.4 ± 2.3 cm.

The characteristics of mothers and newborns for each 8-OHdG tertile are shown in Table 1. A significant proportion of normal-weight mothers were observed in tertile 1 compared with tertile 2 (*p* = 0.035). No other significant differences in clinical characteristics among mothers were observed.

Among the newborns classified by the tertiles for the mothers’ 8-OHdG levels, males were more frequent in tertile 1 than in tertile 2, and caesarean deliveries were more frequent in tertile 3 than in tertile 2. No other significant differences were observed in these groups (Table 1). On the other hand, among newborns classified according to the tertiles of their own 8-OHdG levels, newborns were significantly older in tertile 3 than in tertile 2, and caesarean deliveries were more frequent in tertile 1 than in tertile 2 (Table 1). No other significant differences were detected in the clinical characteristics of newborns in these groups.

### 3.2. Correlation of 8-OHdG Levels between Mothers and Newborns

**Figure 2:**
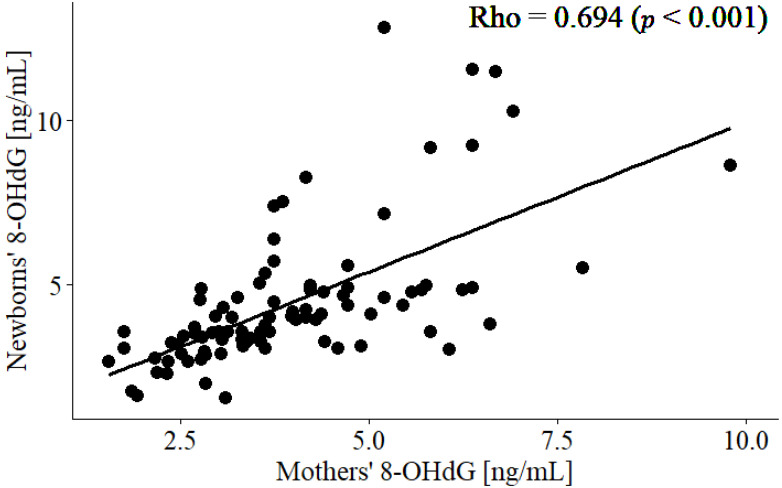
Correlation between the 8-OHdG concentrations of mothers and newborns.

We observed a strong correlation between the 8-OHdG levels of mothers and newborns (*p* < 0.001) (Figure 2). No significant correlations were detected between the 8-OHdG levels of mothers or newborns and the mothers’ age, pregestational weight, pregestational BMI, or the newborns’ age, weight, and height.

### 3.3. Nutritional Differences among Mother Tertiles in the Mothers 8-OHdG Group

Mothers in tertile 3 of the mothers 8-OHdG group consumed significantly lower amounts of calories, carbohydrates, lipids, proteins, and vitamin A, but not vitamin C and E, compared with mothers in tertile 1 (Table 2). No other significant differences were observed in macronutrient and micronutrient consumption by mothers belonging to these tertiles.

### 3.4. Nutritional Differences among the Mother Tertiles in the Newborns 8-OHdG Group

Mothers in tertile 3 of the newborns 8-OHdG group consumed significantly lower amounts of calories, carbohydrates, lipids, proteins, vitamins A and E, but not vitamin C, compared with mothers in tertile 1 (Table 3). Additionally, the mothers in tertile 2 consumed similar amounts of protein to those in tertile 3 (*p* > 0.05) but lower than those in tertile 1 (*p* < 0.05). Lastly, mothers in tertile 2 consumed more vitamin A than those in tertile 3 (*p* < 0.05), but similar to those in tertile 1 (*p* > 0.05). No other significant differences in macronutrient and micronutrient consumption were observed between the mothers in this group (Table 3).

### 3.5. Correlation of Mother Vitamins and Newborns 8-OHdG Levels

**Figure 3:**
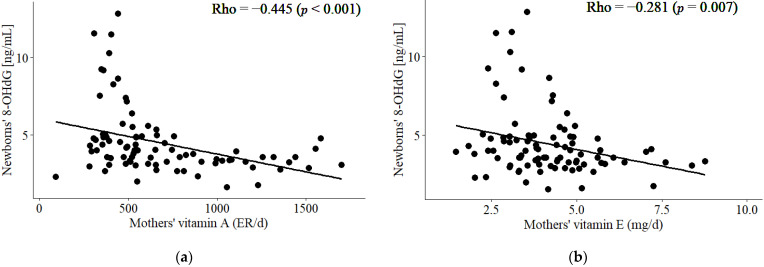
Correlation between vitamin A (**a**) and vitamin E (**b**) consumption by the mothers and 8-OHdG levels in the newborns.

We observed significant correlations between vitamin A and vitamin E consumption by the mothers and 8-OHdG levels in the newborns (*p* < 0.001 and *p* = 0.007, respectively) (Figure 3a,b). A negative but no significant correlation between vitamin C for the mothers and the 8-OHdG levels of newborns was observed (Rho = −0.120, *p* = 0.257).

### 3.6. 8-OHdG Variation by BMI and Vitamin A Consumption

The levels of 8-OHdG in mothers and newborns decreased by a unit in mothers with pregestational obesity. Additionally, the 8-OHdG level in mothers decreased by one unit in tertile 3 of vitamin A consumption, while that in newborns decreased by one and two units in tertiles 2 and 3 of vitamin A consumption, respectively (Table 4). All variations of 8-OHdG were independent of caloric and vitamin intake.

## 4. Discussion

Pregnancy is characterised by increased OS [43], which is associated with metabolic activity and the development of the placenta [44]. DNA is especially susceptible to OS-mediated damage because guanines readily form adducts with FR giving rise to 8-OHdG [18]. During pregnancy, diet might increase or decrease OS, depending on its quality and quantity [17]; for example, the antioxidant properties of vitamins can keep OS in check [45].

In this work, the levels of 8-OHdG found in pregnant women and their newborns were similar to those reported previously [33,46,47,48]. Overweight and obesity in pregnant women have been associated with OS increment [49], OS-mediated DNA damage [50], and increased 8-OHdG levels [51]. However, when we compared the pregestational weight and BMI of mothers with their 8-OHdG levels (classified into tertiles), we did not observe significant associations (Table 1).

We observed that the 8-OHdG levels in mothers and newborns correlated strongly (Figure 2), which is in accordance with our previous study of another OS marker [39]. The source of 8-OHdG in pregnant women and newborns has not been identified yet [52], but Fukushima et al. showed that placental tissue stained positive for 8-OHdG [53]. Since the placenta has imperative functions, such as nutritional support and waste elimination [54], it might be an important, if not the major, source of 8-OHdG [30,52,55].

Maternal diet is an important regulator of OS since carbohydrate, lipid, and amino acid metabolism produces the superoxide radical (O_2_^−^), which through Haber–Weiss and Fenton’s reaction, produces hydrogen peroxide (H_2_O_2_) and the hydroxyl radical (^•^OH), respectively [56]. Consequently, over-nutrition increases OS production [57] and ^•^OH and H_2_O_2_ induce 8-OHdG formation [58,59]. In our study, we expected a significant increment in 8-OHdG when food consumption was higher; however, we observed lower levels of 8-OHdG when the consumption of calories, carbohydrates, lipids, and proteins was higher (Table 2 and Table 3). This effect can only be explained by the antioxidant effects of the diet [60,61]. A study reported that daily fruit intake decreased urinary 8-OHdG secretion in adults [62], hinting that the antioxidant effects of vitamins might limit OS-mediated DNA damage [63]. Multiple studies have reported negative associations between different vitamins and 8-OHdG levels, e.g., plasma vitamin E levels and placental 8-OHdG [64], levels of vitamins E and C and 8-OHdG in the saliva of oral pre-cancer and cancer patients [65], serum vitamin C levels and urinary 8-OHdG [66], and plasma vitamin C levels and lymphocyte 8-OHdG levels in chronic renal disease [67].

To elucidate the effect of vitamins and maternal conditions on 8-OHdG levels, we developed beta analysis regressions. We found that obesity and the intake of vitamin A decreased 8-OHdG levels in mothers and newborns. This effect was maintained even after the analysis was adjusted for caloric intake, and it was more significant in the newborns (Table 4). This finding is contrary to a previous in vitro analysis, where vitamin A increased OS-mediated DNA damage with an increase in 8-oxo-7,8-dihydro-29-deoxyguanosine (8-oxodG) [68], another oxidated derivative of guanine. Nonetheless, vitamin A regulates approximately 532 genes, of which some are involved in antioxidant pathways (Cooper/Zink superoxide dismutase, manganese superoxide dismutase, and glutathione peroxidase 2) [69]. Although vitamin A increased OS-mediated DNA damage in vitro, antioxidant genes might be activated during in vivo OS regulation. The negative association of BMI with 8-OHdG levels might be explained by an increase in the bioavailability of vitamin A in obese people [70,71].

Ascorbic acid and vitamin E have been employed for the treatment of high oxidative conditions such as those found in cardiovascular disease [72], cancer [73], chronic kidney disease [74,75], obesity [76,77], T2D [78], and in smokers [79]. Nonetheless, the effects of vitamin C and E are still controversial since they might play pro-oxidant [80,81,82,83], antioxidant [80,84,85,86], or no [87,88] roles. Specifically, reports on the roles of vitamins E and C in preventing guanine oxidation are mixed [66,81,89]. In this study, vitamins E and C, alone or in combination, failed to reduce 8-OHdG levels. Antioxidant effects of these vitamins have been observed mainly in vitamin-deficient people but not in non-deficient people [66,90]. Interestingly, higher levels of 8-OHdG were observed when the intake of vitamins A and E, but not vitamin C, was lower, and vice versa (Table 2 and Table 3).

Alcohol consumption and smoking are common social practices, especially among young people [91]. In this study, we observed that 10% of women smoked until the first trimester of pregnancy, similar to reports for a Mexican-American population [40]. In some countries, as many as 25% of pregnant women smoke [92]. The proportion of smoking pregnant women in this study was similar to what was reported in a Spanish population [93]. Smoking is a prominent source of FR [94,95], which have deleterious effects on DNA [96,97], giving rise to 8-OHdG [98]. Smoking is associated with cardiovascular disease and cancer [99], and smoking during pregnancy has immediate or midterm effects on the foetus, including preterm pregnancy [100,101,102], low birth weight [102], congenital anomalies [103,104], chronic asthma [92,105,106,107], bronchiolitis [92,108], and psychiatric abnormalities [109,110,111,112]. Unfortunately, we could not follow the newborns of smoking mothers to confirm if they suffered from any of these conditions.

Predisposition to disease during adulthood has been associated with factors pertaining to the mother during the prenatal stage (foetal programming). Accordingly, we hypothesised that a diet rich in vitamins might modify the 8-OHdG levels during pregnancy in the mother–newborn binomial. Previous studies have proposed 8-OHdG as an OS maker in rheumatoid arthritis [48] and atherosclerotic plaque development [27], and as a risk factor for cardiovascular disease [29], metabolic syndrome [28], small-cell lung carcinoma [23], progression of hepatocellular carcinoma [22], and poor glycaemic control of T2D [21]. Consequently, higher levels of 8-OHdG in the progeny due to lower intake of vitamin-rich food by the mother might predispose the child to develop certain diseases in adulthood.

This study had some limitations. Since vitamin consumption was determined by a food frequency questionnaire, it was difficult to establish a precise and objective association between vitamins and 8-OHdG levels since factors like lower absorption rate [113], altered vitamin metabolism [114,115,116], or increased elimination rate [114,117] might modify the bioavailability of vitamins.

## 5. Conclusions

We found a strong correlation between 8-OHdG levels in mothers and newborns. The intake of vitamins A and E, but not C, by the mother might have a greater protective effects against OS-mediated DNA damage in newborns than in mothers.

## Figures and Tables

**Table 1:** Clinical features of mothers and newborns for each 8-OHdG tertile.

Mothers	Mothers 8-OHdG			*p*-Value			Newborns 8-OHdG			*p*-Value		
	Tertile 1 Mean ± SD	Tertile 2 Mean ± SD	Tertile 3 Mean ± SD	a	b	c	Tertile 1 Mean ± SD	Tertile 2 Mean ± SD	Tertile 3 Mean ± SD	a	b	c
Age (years) ^§^	23.1 ± 6.2	24.7 ± 4.9	24.6 ± 4.5	0.250	0.301	1	23.0 ± 5.6	25.4 ± 5.8	24.0 ± 4.0	0.164	0.919	1
Pregestational weight (kg) ^§^	61.0 ± 12.1	66.1 ± 10.5	64.4 ± 10.3	0.167	0.595	1	61.5 ± 11.2	67.3 ± 10.8	62.8 ± 10.7	0.085	1	0.392
Pregestational BMI (kg/m^2^) ^§^	24.9 ± 4.4	27.2 ± 3.9	26.1 ± 3.5	0.156	0.950	1	25.8 ± 4.4	27.1 ± 4.2	25.4 ± 3.4	0.929	1	0.432
Normal-weight (*n*, %) ^§§^	17 (56.7)	8 (26.7)	14 (46.7)	**0.035**	0.605	0.179	16 (53.3)	10 (33.3)	13 (43.3)	0.192	0.605	0.595
Overweight (*n*, %) ^§§^	7 (23.3)	13 (43.3)	10 (33.3)	0.170	0.567	0.595	6 (20.0)	11 (36.7)	13 (43.3)	0.251	0.094	0.792
Obese (*n*, %) ^§§^	6 (20.0)	9 (30.0)	6 (20.0)	0.552	1	0.552	8 (26.7)	9 (30.0)	4 (13.3)	1	0.333	0.209
Smoking (*n*, %)	6 (20.0)	3 (10.0)	0	-	-	-	8 (26.7)	1 (3.3)	0	-	-	-
Multivitamin suppl. (*n*, %) ^§§§^	26 (86.7)	28 (93.3)	28 (93.3)	0.667	0.667	1	27 (90.0)	28 (93.3)	27 (90.0)	1	1	1
**Newborns**												
Gestational age (weeks) ^§^	38.8 ± 1.4	38.5 ± 1.3	38.8 ± 1.1	0.808	1	1	38.8 ± 1.3	38.2 ± 1.4	39.1 ± 0.9	0.450	0.709	**0.026**
Weight (g) ^§^	3286 ± 424	3189 ± 479	3088 ± 389	1	0.468	1	3226 ± 448	3157 ± 415	3180 ± 452	1	1	1
Height (cm) ^§^	49.8 ± 2.4	49.3 ± 2.8	49.3 ± 1.7	1	1	1	49.3 ± 2.9	49.4 ± 2.2	49.6 ± 1.6	1	1	1
Sex (male, *n*, %) ^§§^	18 (60.0)	9 (30)	16 (53.3)	**0.036**	0.794	0.115	14 (46.7)	14 (46.7)	15 (50.0)	1	1	1
Caesarean delivery (*n*, %) ^§§^	6 (20.0)	5 (16.7)	13 (43.3)	1	0.094	**0.047**	11 (36.7)	3 (10.0)	10 (33.3)	**0.030**	1	0.057

^§^ Kruskal–Wallis rank sum test with Dunn Kruskal–Wallis multiple comparison of p-values adjusted with the Bonferroni method. ^§§^ Fisher’s exact Test. ^§§§^ Pearson’s Chi-squared test with Yates’ continuity correction. Tertile comparisons: tertile 1 vs. tertile 2 (a), tertile 1 vs. tertile 3 (b), tertile 2 vs. tertile 3 (c). BMI, body mass index; SD, standard deviation.

**Table 2:** Nutrient differences between mother tertiles in the mothers 8-OHdG group.

Mothers 8-OHdG Tertiles						
	Tertile 1 Median (p25, p75)	Tertile 2 Median (p25, p75)	Tertile 3 Median (p25, p75)	*p*-Value ^§^		
				a	b	c
Calories (kcal/d)	2396 (1712, 3283)	1754 (1551, 2345)	1645 (1429, 1867)	0.202	**0.002**	0.399
Carbohydrates (g/d) % kcal/d	332.4 (252.2, 478.7)	286.4 (249.7, 366.4)	266.3 (216.9, 297.0)	0.633	**0.021**	0.447
	56.9 (53.3, 59.7)	60.3 (55.9, 63.6)	61.0 (54.5, 63.9)	0.053	0.086	1
Lipids (g/d) % kcal/d	69.9 (53.9, 102.6)	48.6 (43.4, 69.5)	45.1 (39.0, 46.9)	0.080	**<0.001**	0.238
	26.1 (24.4, 27.8)	24.1 (22.3, 27.4)	23.5 (21.6, 26.9)	0.233	**0.035**	1
Proteins (g/d) % kcal/d	102.7 (70.9, 150.6)	64.2 (57.6, 94.1)	60.5 (55.8, 83.7)	0.053	**0.002**	0.990
	17.0 (15.0, 18.1)	15.9 (13.8, 16.9)	15.7 (13.7, 18.6)	0.076	0.933	0.666
Vitamin A (ER/d) % adequacy	859.1 (545.7, 1224.8)	521.6 (428.4, 735.5)	489.3 (364.9, 603.7)	0.102	**0.006**	1
	171.8 (109.1, 244.9)	104.3 (85.7, 147.1)	97.9 (72.9, 120.7)	0.102	**0.006**	1
Vitamin C (mg/d) % adequacy	138.2 (113.4, 222.7)	198.3 (147.9, 226.2)	157.0 (137.2, 172.8)	0.111	1	0.195
	230.2 (189.1, 371.2)	330.6 (246.6, 377.0)	261.6 (228.5, 288.0)	0.111	1	0.195
Vitamin E (mg/d) % adequacy	4.26 (3.31, 5.13)	4.20 (3.51, 4.96)	3.69 (3.03, 4.39)	1	0.296	0.362
	35.4 (27.6, 42.7)	35.0 (29.3, 41.3)	30.8 (25.3, 36.6)	1	0.296	0.362

^§^ Kruskal–Wallis rank sum test with Dunn Kruskal–Wallis multiple comparison of p-values adjusted with the Bonferroni method. Tertile comparisons: tertile 1 vs. tertile 2 (a), tertile 1 vs. tertile 3 (b), tertile 2 vs. tertile 3 (c). p, percentile.

**Table 3:** Nutrient differences by mother tertiles in the newborns 8-OHdG group.

Newborns 8-OHdG Tertile						
	Tertile 1 Median (p25, p75)	Tertile 2 Median (p25, p75)	Tertile 3 Median (p25, p75)	*p*-Value ^§^		
				a	b	c
Calories (kcal/d)	2446 (1787, 3283)	1772 (1539, 2344)	1616 (1334, 1816)	0.068	**<0.001**	0.284
Carbohydrates (g/d) % kcal/d	372.4 (269.0, 478.7)	281.9 (245.2, 352.7)	256.3 (201.1, 296.2)	0.076	**<0.001**	0.406
	57.4 (54.4, 60.2)	59.9 (56.9, 63.6)	60.6 (54.4, 62.6)	0.406	0.650	1
Lipids (g/d) % kcal/d	73.3 (52.3, 102.8)	51.43 (42.24, 69.35)	44.85 (41.10, 47.22)	0.0912	**<0.001**	0.273
	25.4 (23.3, 27.3)	23.79 (22.28, 27.50)	24.28 (22.96, 28.86)	0.754	1	1
Proteins (g/d) % kcal/d	124.2 (77.2, 157.9)	68.3 (58.4, 98.9)	61.0 (56.3, 72.7)	**0.034**	**<0.001**	0.455
	17.1 (14.9, 18.1)	15.9 (13.8, 17.7)	15.1 (13.8, 17.4)	0.334	0.243	1
Vitamin A (ER/d) % adequacy	853.3 (545.1, 1191)	590.4 (479.0, 964.4)	428.5 (364.9, 539.0)	0.499	**<0.001**	**0.015**
	170.6 (109.0, 238.2)	118.0 (95.8, 192.8)	85.7 (72.9, 107.8)	0.499	**<0.001**	**0.015**
Vitamin C (mg/d) % adequacy	202.3 (124.6, 248.7)	161.8 (132.0, 226.2)	154.0 (129.6, 172.2)	1	0.167	0.576
	337.2 (207.7, 414.5)	269.8 (220.0, 377.0)	256.7 (216.0, 287.0)	1	0.167	0.576
Vitamin E (mg/d) % adequacy	4.71 (3.65, 5.43)	3.87 (3.33, 4.77)	3.56 (2.90, 4.32)	0.179	**0.005**	0.655
	39.3 (30.4, 45.2)	32.3 (27.7, 39.8)	29.6 (24.2, 35.9)	0.179	**0.005**	0.655

^§^ Kruskal–Wallis rank sum test with Dunn Kruskal–Wallis multiple comparison of p-values adjusted with the Bonferroni method. Tertile comparisons: tertile 1 vs. tertile 2 (a), tertile 1 vs. tertile 3 (b), tertile 2 vs. tertile 3 (c).

**Table 4:** Multiple regression analysis for the 8-OHdG levels in mothers and newborns.

Parameter	Mothers 8-OHdG (ng/mL) β (95% CI) ^a^	*p*-Value	Newborns 8-OHdG (ng/mL) β (95% CI) ^a^	*p*-Value
Pregestational BMI				
Normal-weight	Reference		Reference	
Overweight	−0.002 (−0.64 to 0.63)	0.995	0.048 (−0.56 to 0.66)	0.874
Obesity	−1.04 (−1.78 to −0.29)	**0.007**	−0.977 (−1.69 to −0.26)	**0.008**
Vitamin A (ER/d)				
Tertile 1 (91 to 475)	Reference		Reference	
Tertile 2 (485 to 745)	−0.123 (−0.90 to 0.66)	0.753	−1.15 (−1.90 to −0.41)	**0.003**
Tertile 3 (757 to 4028)	−1.26 (−2.28 to −0.24)	**0.016**	−2.17 (−3.15 to −1.19)	**<0.001**

^a^ Quantile regression model adjusted by caloric intake. CI, confidence interval.

## Data Availability

The set of raw data analysed is available for other research studies upon application.
